# Unchanged type 1 metabotropic glutamate receptor availability in patients with Alzheimer's disease: A study using ^11^C-ITMM positron emission tomography

**DOI:** 10.1016/j.nicl.2019.101783

**Published:** 2019-03-16

**Authors:** Kenji Ishibashi, Yoshiharu Miura, Jun Toyohara, Kiichi Ishiwata, Kenji Ishii

**Affiliations:** a Research Team for Neuroimaging, Tokyo Metropolitan Institute of Gerontology, Tokyo, Japan; b Department of Neurology, Tokyo Metropolitan Cancer and Infectious Diseases Center Komagome Hospital, Tokyo, Japan; c Institute of Cyclotron and Drug Discovery Research, Southern Tohoku Research Institute for Neuroscience, Fukushima, Japan; d Department of Biofunctional Imaging, Fukushima Medical University, Fukushima, Japan

**Keywords:** Type 1 metabotropic glutamate receptor, Alzheimer's disease, PET, ^11^C-ITMM

## Abstract

Imaging of type 1 metabotropic glutamate receptor (mGluR1) has recently become possible using positron emission tomography (PET). To date, little evidence exists on the role of mGluR1 in the pathophysiology of Alzheimer's disease (AD). We aimed to examine mGluR1 availability in patients with AD. Ten patients with AD (78.9 ± 5.9 years) and 12 age-matched volunteers (74.6 ± 2.6 years) underwent PET using an mGluR1 radiotracer. All patients were anti-dementia drug-naive. Volumes-of-interest were placed on the anterior and posterior lobes and vermis in the cerebellum and frontal, parietal, and temporal cortices. The binding potential (BP_ND_) was calculated to estimate mGluR1 availability, and partial volume correction was applied to the BP_ND_ values. Mini Mental State Examination (MMSE) scores were also obtained (22.0 ± 4.8). No significant difference was observed in BP_ND_ between the AD and control groups in the anterior lobe (p = .30), posterior lobe (p = .95), vermis (p = .96), frontal cortex (p = .61), parietal cortex (p = .59), or temporal cortex (p = .27). No significant correlation was observed between BP_ND_ and MMSE scores in the anterior lobe (p = .59), posterior lobe (p = .35), vermis (p = .92), frontal cortex (p = .78), parietal cortex (p = .83), or temporal cortex (p = .82). In conclusions, this study suggests that mGluR1 availability is unchanged in the relatively early stage of AD. However, because regional mGluR1 availability may change with the progression of AD, further longitudinal follow-up is necessary.

## Highlights

• Metabotropic glutamate receptors (mGluR) can be affected in AD.
• Little evidence exists on the role of type 1 mGluR (mGluR1) in AD.
• We examined mGluR1 availability in patients with AD.
• mGluR1 availability was unchanged in the relatively early stage of AD.

## 1 Introduction

Imaging of type 1 metabotropic glutamate receptor (mGluR1) in living human brains became possible in the early 2010s using positron emission tomography (PET) (Toyohara et al., 2013a; Toyohara et al., 2013b). In concordance with the existing knowledge that mGluR1 is predominantly expressed on the dendrites of Purkinje cells facing the synaptic terminals of parallel and climbing fibers (Ferraguti et al., 2008), the first PET imaging of mGluR1 in human volunteers showed that its availability is higher in the cerebellar cortex than the cerebral cortex (Toyohara et al., 2013a; Toyohara et al., 2013b). These findings suggest that alterations in cerebellar mGluR1 expression are closely linked to the pathophysiology of Purkinje cells. As shown in animal studies (Aiba et al., 1994; Ichise et al., 2000; Notartomaso et al., 2013; Sillevis Smitt et al., 2000), the reduction in cerebellar mGluR1 availability was recently confirmed to possibly be associated with the development of cerebellar ataxia in human patients using PET imaging of mGluR1 (Ishibashi et al., 2016; Ishibashi et al., 2017).

Compared to the number of *in vivo* studies focusing on mGluR1 in the cerebellum, very few *in vivo* studies have investigated the role of mGluR1 in the cerebrum, probably because its expression in the cerebrum is relatively poor. However, because metabotropic glutamate receptors can be involved in the regulation of neuronal excitability and synaptic plasticity, cerebral mGluR1 playing a critical role in the development and protection of neurons in some neurological and psychiatric disorders is unsurprising (Ferraguti et al., 2008). Ostapchenko and colleagues recently showed that mGluR1 expression in the cerebrum can increase in a mouse model of Alzheimer's disease (AD), probably because of exposure to Aβ oligomers (Ostapchenko et al., 2013). In contrast to their findings, Albasanz and colleagues assessed the postmortem brains of 10 patients with common-form dementia with Lewy bodies (DLB) (*i.e.*, DLB accompanied by AD-related pathology), and found that mGluR1 expression in the cerebrum can decrease with progression of the pathological stages of AD (Albasanz et al., 2005). This difference between the two aforementioned studies may indicate that cerebral mGluR1 expression in AD depends on its clinical stage and/or coexisting diseases such as DLB. To advance the understanding of the exact role of mGluR1 in AD, *in vivo* imaging of mGluR1 in patients with AD is necessary. Hence, we examined mGluR1 availability across the brain in living patients with AD, using *N*-[4-[6-(isopropylamino)pyrimidin-4-yl]-1,3-thiazol-2-yl] -4-^11^C-methoxy-*N*-methylbenzamide (^11^C-ITMM) and PET.

## 2 Materials and methods

### 2.1 Research participants

**Fig. 1:**
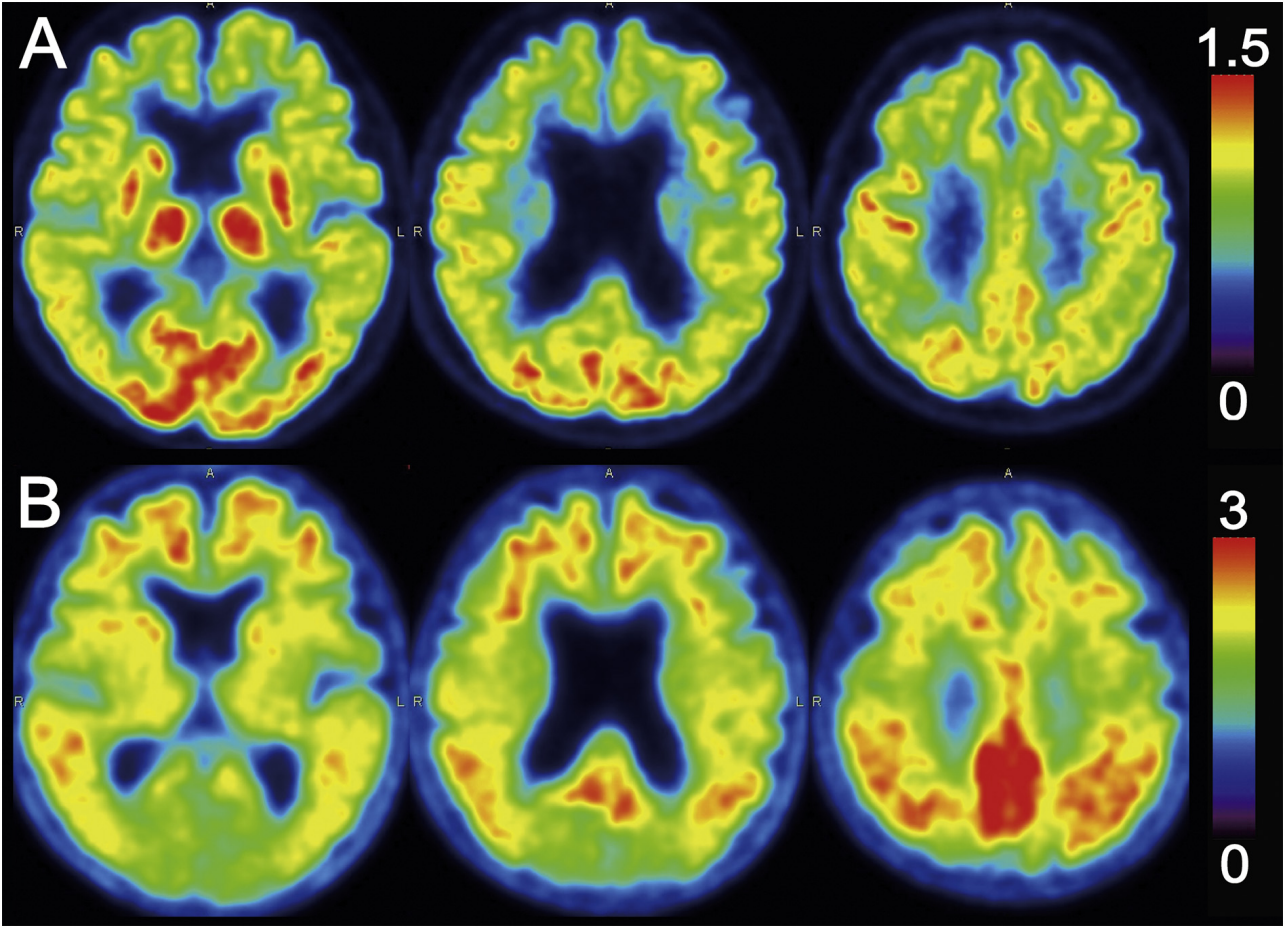
A representative case of ^18^F-FDG (A) and ^11^C-PiB (B) images. ^18^F-FDG and ^11^C-PiB images were normalized using the cerebellum as a reference region (*i.e.*, cerebellar uptake was set as one), and are displayed in axial sections. Especially in the posterior cingulate and precuneus cortices, ^18^F-FDG uptake decreased (A) and ^11^C-PiB uptake increased (B), showing a pattern characteristic of Alzheimer's disease. This case was from the patient 6 in Table 1.

**Fig. 2:**
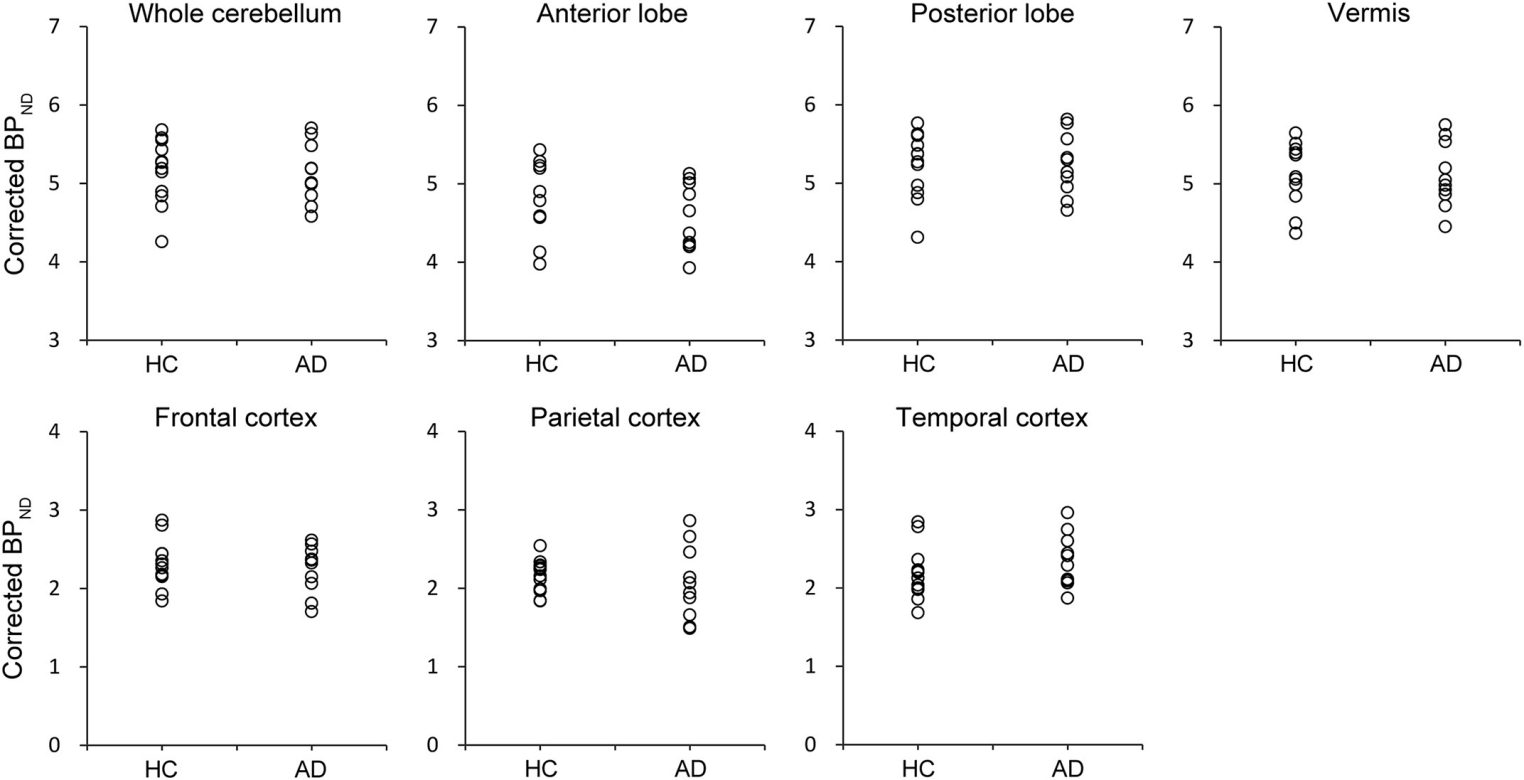
BP_ND_ across the brain in the Alzheimer's disease (AD) and healthy control (HC) groups. The corrected BP_ND_ values in the AD (n = 10) and healthy control (n = 12) groups were plotted as open circles in the whole cerebellum, anterior lobe, posterior lobe, vermis, frontal cortex, parietal cortex, and temporal cortex. No significant difference was observed between the two groups across the brain.

This study was conducted in accordance with the Declaration of Helsinki and was approved by the Ethics Committee of the Tokyo Metropolitan Institute of Gerontology (H26-81). Written informed consent was obtained from all participants who underwent ^11^C-ITMM PET and magnetic resonance imaging (MRI) scans. The participants comprised 10 patients (3 men and 7 women; 78.9 ± 5.9 years) who visited a neurologist complaining of the development of memory loss, and 12 age-matched volunteers (5 men and 7 women; 74.6 ± 2.6 years). All patients underwent ^18^F-fluorodeoxyglucose (^18^F-FDG) PET, which provides an index of regional glucose metabolism, and exhibited a pattern characteristic of AD; ^18^F-FDG uptake decreased in the posterior cingulate, precuneus, and/or temporoparietal cortices (S-Fig. 1 in a supplementary file). The subsequent ^11^C-Pittsburgh compound B (^11^C-PiB) PET yielded positive findings for Aβ accumulation in all patients (S-Fig. 2 in a supplementary file). All patients were anti-dementia drug-naive at the time of ^11^C-ITMM PET, and the Mini Mental State Examination (MMSE) and Clinical Dementia Rating scale (CDR) scores of the patients were 22.0 ± 4.8 and 0.8 ± 0.3 (0.5 or 1), respectively (Table 1). Although some of the patients might be in the early stage of the AD spectrum at the time of ^11^C-ITMM PET, all patients were confirmed to fulfill the AD criteria (American-Psychiatric-Association, 1994) in the regular follow-up examinations after ^11^C-ITMM PET. All volunteers were deemed healthy based on their medical history, the results of physical and neurological examinations performed by a neurologist, and MRI findings. The MMSE and CDR scores of the volunteers were 29.3 ± 1.0 (score 30: n = 6, score 29: n = 4, score 28: n = 1, score 27: n = 1) and 0, respectively (Table 1). Data are expressed as mean ± SD.

**Table 1:** Characteristics of all participants at the time of ^11^C-ITMM PET.

Group		Age	Sex	Duration (y)	MMSE	CDR
Patient	1	74	F	1	19	1
	2	85	M	3	14	1
	3	83	M	1	27	0.5
	4	74	M	2	28	0.5
	5	83	F	1	19	1
	6	82	F	1	28	0.5
	7	75	F	2	23	1
	8	69	F	2	24	0.5
	9	77	F	1	18	1
	10	87	F	1	20	1
	n = 10	78.9 ± 5.9⁎	M: 3, F: 7		22.0 ± 04.8⁎⁎	0.8 ± 0.3⁎⁎
Control	n = 12	74.6 ± 2.6⁎	M: 5, F: 7		29.3 ± 1.0⁎⁎	0⁎⁎

The data represent mean ± standard deviation.

MMSE: Mini Mental State Examination.

CDR: Clinical Dementia Rating scale.

⁎ Not significant (p > .05) between patients and controls using a two-tailed t-test.

⁎⁎ Significant (p < .001) between patients and controls using a two-tailed t-test.

### 2.2 PET scanning and MRI acquisition

The radioligand, ^11^C-ITMM, was synthesized as described previously (Toyohara et al., 2013a), and was used to measure mGluR1 availability. The PET scanning was performed at the Tokyo Metropolitan Institute of Gerontology using the Discovery PET/CT 710 scanner (GE Healthcare, Milwaukee, WI, USA) in three-dimensional mode. The in-plane and axial resolutions for full width at half maximum were 4.52 and 4.83 mm, respectively. Images with 47 slices were obtained with a voxel size of 2 × 2 × 3.27 mm and a matrix size of 128 × 128. A computed tomography transmission scan was performed to obtain data for measured attenuation correction. After a bolus injection of ^11^C-ITMM, dynamic emission data were acquired for 70 min. For the AD group, the injection doses and molar activities at the time of injection were 498.9 ± 53.5 MBq and 117.7 ± 41.8 GBq/μmol, respectively (mean ± SD). For the control group, the corresponding values were 542.7 ± 67.1 MBq and 84.4 ± 32.1 GBq/μmol, respectively. The frame arrangements for the dynamic PET data were 20 s × 3 frames, 30 s × 3 frames, 60 s × 5 frames, 150 s × 5 frames, and 300 s × 10 frames. Data were reconstructed after correction for decay, attenuation, and scatter.

The MRI scanning was performed using the Discovery MR750w 3.0 T scanner (GE Healthcare, Milwaukee, WI, USA) in three-dimensional mode (spoiled gradient-recalled images: repetition time, 7.648 ms; echo time, 3.092 ms; matrix size, 196 × 256 × 256; and voxel size, 1.2 × 1.0547 × 1.0547 mm). The PET and MRI data were processed using the FMRIB Software Library version 5.0.4 (FSL; Oxford University, Oxford, UK).

### 2.3 Volumes-of-interest and PET image processing

The cerebellar subregions, three cortical lobes (frontal, parietal, and temporal) and white matter were defined as volumes-of-interest (VOIs). The VOIs for the anterior and posterior lobes and vermis in the cerebellum were selected from the probabilistic cerebellar atlas included in the FSL. The VOI for the whole cerebellum was created by combining the VOIs of the cerebellar subregions. The VOIs for the three cortices were selected from the Montreal Neurological Institute (MNI) structural atlas included in the FSL. The VOI for the white matter was created in the MNI space by combining the VOIs of the superior corona radiata, superior longitudinal fasciculus, splenium of the corpus callosum, and pons to include sufficient volume for the subsequent kinetic analysis. The VOIs of the superior corona radiata, superior longitudinal fasciculus, and splenium of the corpus callosum were sampled from the JHU DTI-based white matter atlas included in the FSL, and the pons VOI was manually drawn in the MNI space. These VOIs in the MNI space were then transformed into native space and used for the subsequent kinetic analysis.

Time-activity data were extracted from the anatomically defined VOIs on the PET images and imported into the PMOD PKIN version 3.4 (PMOD Technologies, Zurich, Switzerland). First, the time-activity curves were fitted using the Simplified Reference Tissue Model (SRTM) to provide an estimation of k2′, the rate constant for the transfer of the radioligand from the reference region to the plasma (Lammertsma and Hume, 1996). We used the white matter as a reference region because mGluR1 expression is negligible in the medulla (Li and Huang, 2014; Martin et al., 1992; Yamasaki et al., 2014), and computed the k2′ value from the high-activity region, the whole cerebellum VOI, for each subject. The time-activity curves were then refitted with the SRTM2 (Wu and Carson, 2002), and the computed k2′ values were applied to all VOIs for each subject. The binding potential (BP_ND_) was then calculated to estimate mGluR1 availability. Additionally, BP_ND_ maps were generated in native space using the PMOD PXMOD version 3.4 and the SRTM2 (Wu and Carson, 2002).

### 2.4 Partial volume correction of BP_ND_

Patients with AD and elderly volunteers typically exhibit brain atrophy, which results in a specific reduction of BP_ND_ values because of a partial volume effect. To overcome this problem, a two-compartmental partial volume correction was applied to the BP_ND_ values of all of the participants (Meltzer et al., 1990). First, partial volume maps were generated for each class (gray matter, white matter, and cerebrospinal fluid) from the structural MRI in native space. In the partial volume maps, each voxel had a value ranging from 0 to 1, which represented the proportion of each class. A correction map of the brain parenchyma was then created through the addition of the gray and white matter partial volume maps and convolution with a Gaussian filter to approximate the corresponding PET spatial resolution. This correction map was masked with each VOI, and a correction factor was computed for each VOI. Time-activity data extracted from each VOI were divided by the corresponding correction factor to yield the corrected time-activity data. The corrected BP_ND_ values were then calculated using the corrected time-activity data with the SRTM, as previously described (Lammertsma and Hume, 1996; Wu and Carson, 2002). The BP_ND_ maps were also corrected using the correction maps.

### 2.5 Data analysis

The differences in variables between the AD and control groups were tested using a two-tailed t-test. The relationships between BP_ND_ and MMSE scores in each VOI were tested using correlation analysis with a two-tailed test. All statistical analyses were conducted using the SPSS version 22 (IBM, Armonk, NY, USA). Statistical significance was set at p < .05.

## 3 Results

The characteristics of all participants at the time of ^11^C-ITMM PET are summarized in Table 1, showing that the patients were predominantly distributed in the relatively early stage of the AD spectrum. A representative case is displayed in Fig. 1 (patient 6 in Table 1), where ^18^F-FDG and ^11^C-PiB images showed the pattern characteristic of AD.

Plots of the corrected BP_ND_ values in each region are shown in Fig. 2. For the AD group, the corrected BP_ND_ values were 5.13 ± 0.38, 4.57 ± 0.43, 5.24 ± 0.40, 5.11 ± 0.42, 2.24 ± 0.31, 2.07 ± 0.47, and 2.36 ± 0.34 in the whole cerebellum, anterior lobe, posterior lobe, vermis, frontal cortex, parietal cortex, and temporal cortex, respectively (mean ± SD). For the control group, the corresponding values were 5.15 ± 0.42, 4.77 ± 0.46, 5.23 ± 0.42, 5.10 ± 0.40, 2.31 ± 0.31, 2.16 ± 0.21, and 2.19 ± 0.34, respectively. No significant difference was observed between the AD and control groups in the whole cerebellum (p = .91), anterior lobe (p = .30), posterior lobe (p = .95), vermis (p = .96), frontal cortex (p = .61), parietal cortex (p = .59), or temporal cortex (p = .27).

**Fig. 3:**
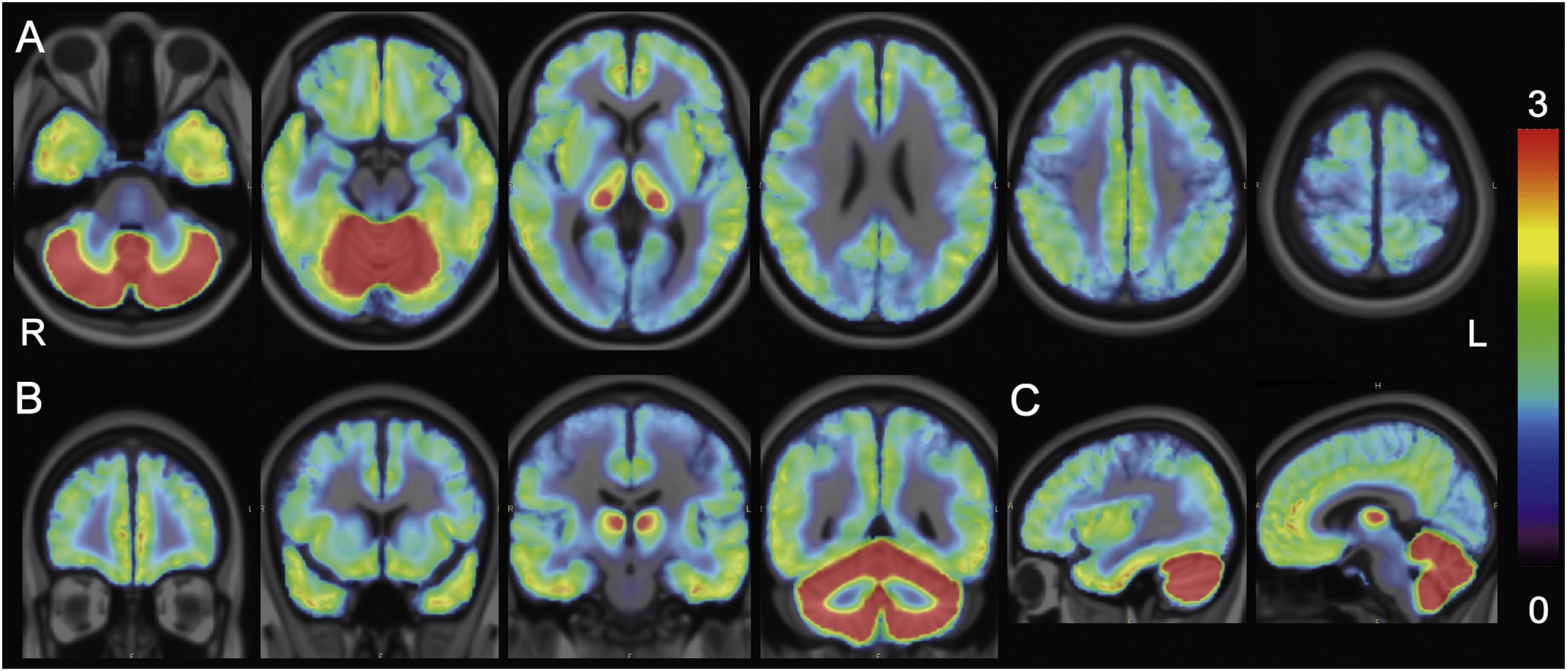
BP_ND_ maps in the Alzheimer's disease (AD) group. Corrected BP_ND_ maps of 10 patients with AD were averaged in the Montreal Neurological Institute (MNI) space and displayed on the MNI brain in the axial (A), coronal (B), and sagittal (C) views. The rainbow-colored scale represents the magnitude of the corrected BP_ND_ values.

**Fig. 4:**
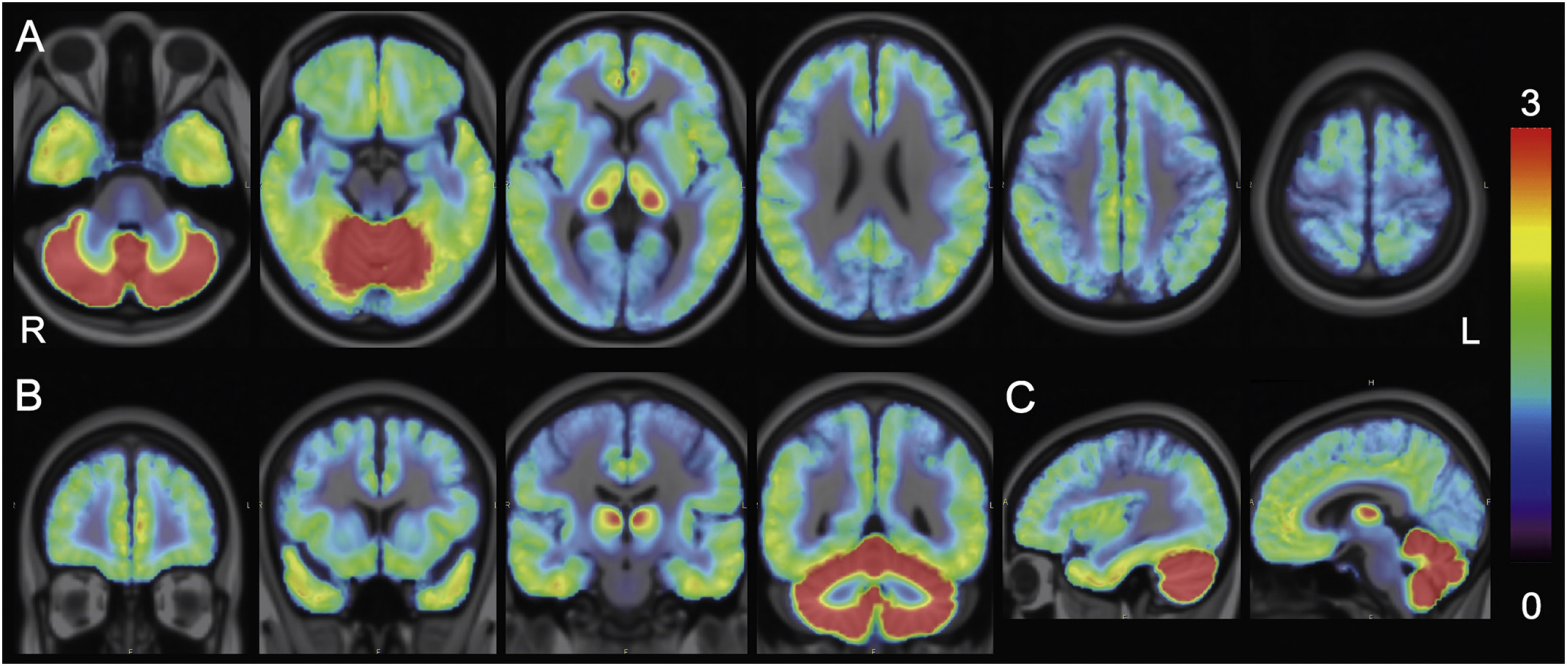
BP_ND_ maps in the healthy control (HC) group. Corrected BP_ND_ maps of 12 healthy volunteers were averaged in the Montreal Neurological Institute (MNI) space and displayed on the MNI brain in the axial (A), coronal (B), and sagittal (C) views. The rainbow-colored scale represents the magnitude of the corrected BP_ND_ values.

The corrected BP_ND_ maps were averaged and visually inspected to identify any regional differences in the cortical BP_ND_ values between the AD (Fig. 3) and control (Fig. 4) groups. In line with the results from the VOI analyses (Fig. 2), the distribution of BP_ND_ values was similar between the two groups across the cortical areas.

No significant correlation was observed between the corrected BP_ND_ values and MMSE scores in the whole cerebellum (r = 0.33, p = .35), anterior lobe (r = 0.19, p = .59), posterior lobe (r = 0.33, p = .35), vermis (r = 0.04, p = .92), frontal cortex (r = 0.10, p = .78), parietal cortex (r = −0.08, p = .83), or temporal cortex (r = 0.08, p = .82).

## 4 Discussion

The objectives of this study were to assess whether mGluR1 availability differs between the AD and control groups and whether mGluR1 availability and MMSE scores are correlated in patients with AD. Because patients with AD and elderly volunteers are susceptible to a partial volume effect, we applied partial volume correction to the BP_ND_ values. Although we showed results only from the corrected BP_ND_ values, regardless of partial volume correction (*i.e.*, corrected or uncorrected BP_ND_ values), we found no difference in mGluR1 availability between the AD and control groups across the brain, and no correlation between mGluR1 availability and MMSE scores.

Metabotropic glutamate receptors are likely to be involved in regulating excitatory neurotransmission and synaptic plasticity, which are affected in AD (Cosgrove et al., 2011; Luscher and Huber, 2010). Eight subtypes of metabolic glutamate receptors, mGluR1 to mGluR8, have been identified and classified into three subgroups. Group I comprises mGluR1 and mGluR5. Unlike mGluR1, mGluR5 expression is rich in the cerebrum (Varnas et al., 2018), and a larger number of studies have investigated the role of mGluR5 in AD-related cognitive dysfunction, suggesting that it is a promising target for AD treatment (Caraci et al., 2018). Conversely, sparse evidence exists on the role of cerebral mGluR1 in the pathophysiology of AD, although several studies have investigated the contribution of cerebellar mGluR1 to cerebellar ataxia (Aiba et al., 1994; Ichise et al., 2000; Ishibashi et al., 2016; Ishibashi et al., 2017; Notartomaso et al., 2013; Sillevis Smitt et al., 2000). Two studies have investigated the relationship between mGluR1 and AD; one study found increased expression of cerebral mGluR1 in a mouse model of AD (Ostapchenko et al., 2013) and the other found decreased expression of cerebral mGluR1 in postmortem brains with DLB accompanied by AD related-pathology (Albasanz et al., 2005). The results of the present study, the first PET study on living patients with AD, do not support either of the two aforementioned studies.

The present study had some limitations. The number of patients with AD was relatively small, and the patients were predominantly distributed in the early stage of the AD spectrum based on the clinical course of each patient and results from ^18^F-FDG, ^11^C-PiB and voxel-based morphometry analyses (see a supplementary file). Therefore, one cannot deny the possibility that regional mGluR1 availability may change with the progression of the clinical stages of AD. Further follow-up of the AD patients in this study will be needed to elucidate this issue.

## Appendix A Supplementary data

Supplementary material Image 1
