# Supplementary material for: Unchanged type 1 metabotropic glutamate receptor availability in patients with Alzheimer's disease: A study using 11C-ITMM positron emission tomography
Source: Neuroimage Clin. 2019 Mar 16;22:101783. doi: 10.1016/j.nicl.2019.101783 (PMC6434168; doi:10.1016/j.nicl.2019.101783)
Supplement: Supplementary file 1 — Supplementary material [file mmc1.pdf]

A supplementary file

Unchanged type 1 metabotropic glutamate receptor availability in patients with Alzheimer's disease: A study using ^{11}C-ITMM positron emission tomography

Kenji Ishibashi[1,2*], Yoshiharu Miura[2], Jun Toyohara[1], Kiichi Ishiwata[1,3,4], and Kenji Ishii[1]

[1]Research Team for Neuroimaging, Tokyo Metropolitan Institute of Gerontology, Tokyo, Japan
[2]Department of Neurology, Tokyo Metropolitan Cancer and Infectious Diseases Center Komagome Hospital, Tokyo, Japan
[3]Institute of Cyclotron and Drug Discovery Research, Southern Tohoku Research Institute for Neuroscience, Fukushima, Japan
[4]Department of Biofunctional Imaging, Fukushima Medical University, Fukushima, Japan

Correspondence to: Kenji Ishibashi, MD, PhD, Research Team for Neuroimaging, Tokyo Metropolitan Institute of Gerontology, 35-2 Sakae-cho, Itabashi-ku, Tokyo 173-0015, Japan
Telephone: +81 3-3964-3241, Fax: +81 3-3964-1148
E-mail: ishibashi@pet.tmig.or.jp
E-mail of the others: ymiura@cick.jp (Yoshiharu Miura), toyohara@pet.tmig.or.jp (Jun Toyohara), ishiwata@pet.tmig.or.jp (Kiichi Ishiwata), ishii@pet.tmig.or.jp (Kenji Ishii)

S-Figure 1. Whole-brain voxelwise analysis: comparison of ^{18}F-FDG uptake between the patient (n = 10) and control (n = 26) group.

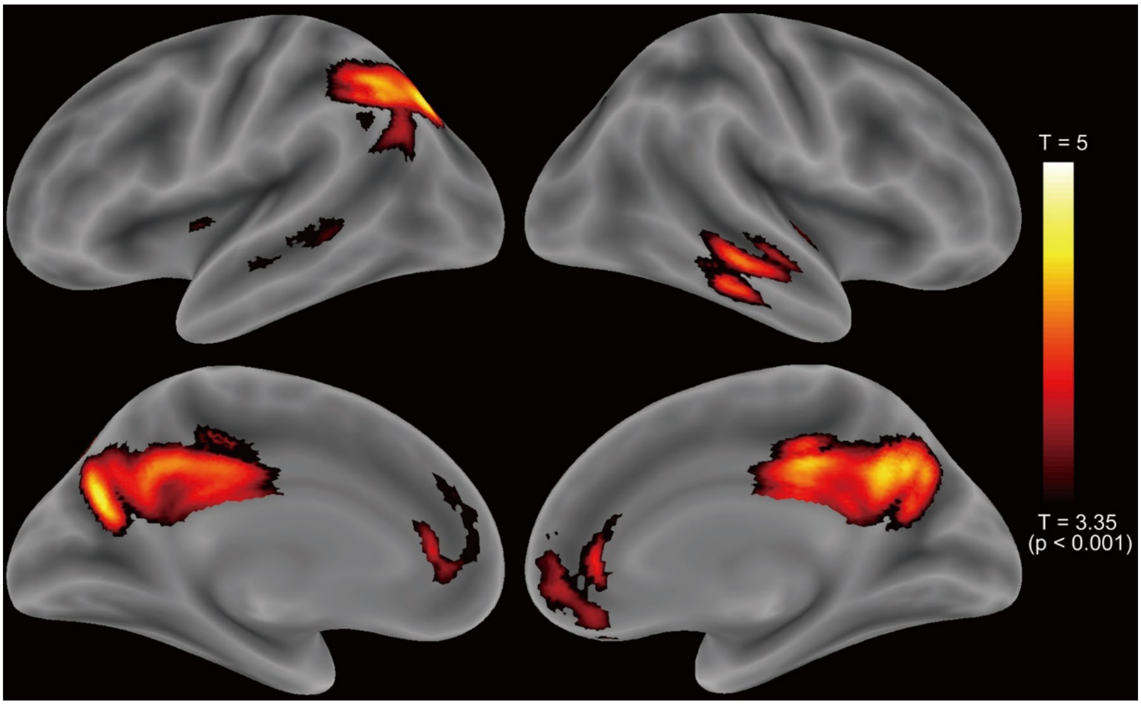

The patient group (n = 10) in the main manuscript underwent both ^{11}C-ITMM and ^{18}F-FDG PET. Meanwhile, the control group (n = 12) in the main manuscript underwent only ^{11}C-ITMM PET. Therefore, we set another control group (n = 26, 76.2 ± 3.6 years) to assess the regions where ^{18}F-FDG uptake in the patient group decreased.

^{18}F-FDG images were nonlinearly transformed into the standard space, and smoothed with an isotropic Gaussian kernel with a sigma of 6 mm. The whole-brain voxelwise analysis was then performed to compare ^{18}F-FDG distribution between the patient and control groups, using Statistical Parametric Mapping version 12 and bspmview (http://www.bobspunt.com/bspmview). A two-sample t test and "proportional scaling" for the global normalization method were specified. A statistical t map of "control group: 1 and patient group: -1" contrast was calculated. The threshold was set at $p < 0.001$ uncorrected ($T > 3.35$).

The results from the whole-brain voxelwise analysis are displayed above, showing the clusters where ^{18}F-FDG uptake in the patient group was lower than that in the control group. The voxels with the higher statistical values extended to the posterior cingulate, precuneus, and temporoparietal cortices, showing a pattern characteristic of AD.

S-Figure 2. ^{11}C-PiB images in all patients (n = 10).

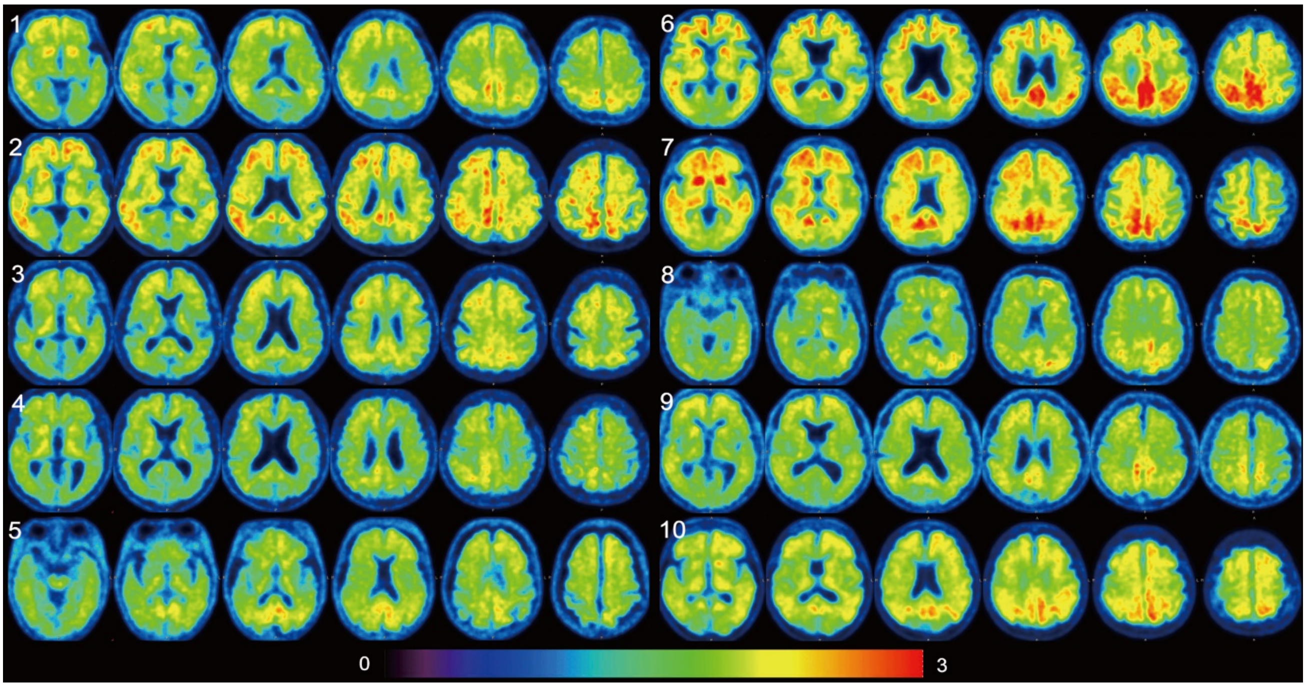

All patients (n = 10) in the main manuscript underwent ^{11}C-PiB PET. ^{11}C-PiB images were normalized using the cerebellum as a reference region (i.e., cerebellar uptake was set as one).

^{11}C-PiB images in each patient (numbers from 1 to 10 in Table 1 of the main manuscript) are displayed above in axial sections. All patients were visually comfirmed to have positive findings for Aβ accumulation. The rainbow scale represents the magnitude of normalized ^{11}C-PiB uptake.

S-Figure 3. Voxel-based morphometry (VBM) analysis between the patient (n = 10) and control (n = 12) group

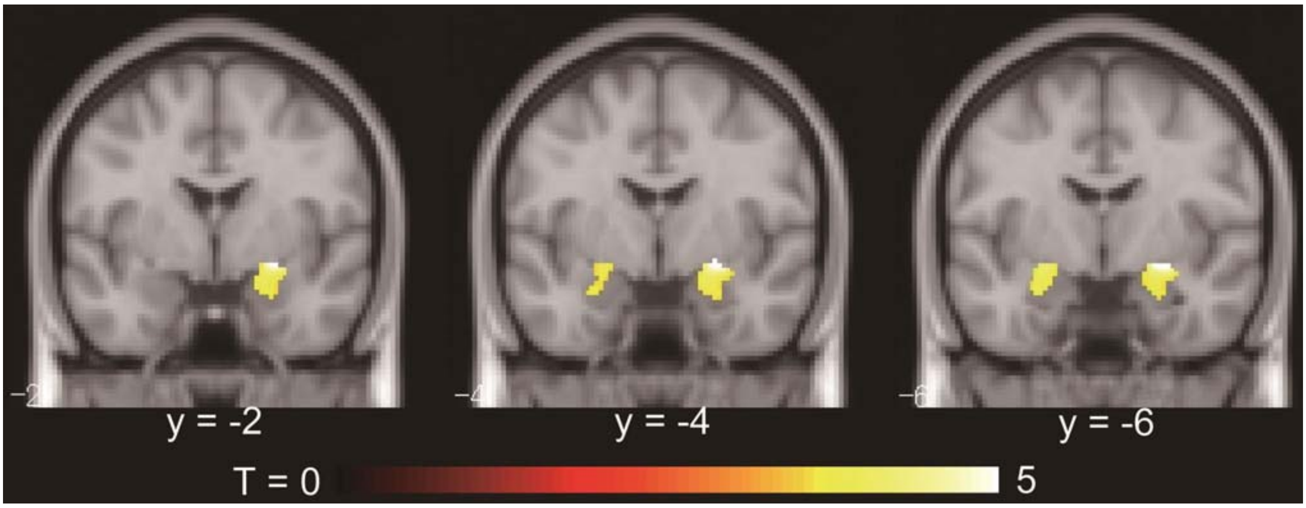

The VBM analysis was performed to assess the voxelwise differences in the cerebral cortical volume between the patient (n = 10) and control (n = 12) groups.

The structural T1-weighted images were processed using FSLVBM version 1.1 (https://fsl.fmrib.ox.ac.uk/fsl/fslwiki/FSLVBM/UserGuide) and Statistical Parametric Mapping version 12. A statistical t map of "control group: 1 and patient group: -1" contrast was calculated. The threshold was set at $p < 0.001$ uncorrected ($T > 3.55$).

The results from the VBM analysis are displayed above, showing the clusters where the cerebral cortical volume in the patient group was lower than that in the control group. The voxels with the higher statistical values extended to the medial temporal lobe around the hippocampus, showing a pattern characteristic of AD.
